# Single-dose azithromycin for infant growth in Burkina Faso: Prespecified secondary anthropometric outcomes from a randomized controlled trial

**DOI:** 10.1371/journal.pmed.1004345

**Published:** 2024-01-23

**Authors:** Ali Sié, Mamadou Ouattara, Mamadou Bountogo, Clarisse Dah, Thierry Ouedraogo, Valentin Boudo, Elodie Lebas, Huiyu Hu, Benjamin F. Arnold, Kieran S. O’Brien, Thomas M. Lietman, Catherine E. Oldenburg

**Affiliations:** 1 Centre de Recherche en Santé de Nouna, Nouna, Burkina Faso; 2 Francis I Proctor Foundation, University of California, San Francisco, United States of America; 3 Department of Ophthalmology, University of California, San Francisco, United States of America; 4 Institute for Global Health Sciences, University of California, San Francisco, United States of America; 5 Department of Epidemiology & Biostatistics, University of California, San Francisco, United States of America; The Hospital for Sick Children, CANADA

## Abstract

**Background:**

Antibiotic use during early infancy has been linked to childhood obesity in high-income countries. We evaluated whether a single oral dose of azithromycin administered during infant-well visits led to changes in infant growth outcomes at 6 months of age in a setting with a high prevalence of undernutrition in rural Burkina Faso.

**Methods and findings:**

Infants were enrolled from September 25, 2019, until October 22, 2022, in a randomized controlled trial designed to evaluate the efficacy of a single oral dose of azithromycin (20 mg/kg) compared to placebo when administered during well-child visits for prevention of infant mortality. The trial found no evidence of a difference in the primary endpoint. This paper presents prespecified secondary anthropometric endpoints including weight gain (g/day), height change (mm/day), weight-for-age Z-score (WAZ), weight-for-length Z-score (WLZ), length-for-age Z-score (LAZ), and mid-upper arm circumference (MUAC). Infants were eligible for the trial if they were between 5 and 12 weeks of age, able to orally feed, and their families were planning to remain in the study area for the duration of the study. Anthropometric measurements were collected at enrollment (5 to 12 weeks of age) and 6 months of age. Among 32,877 infants enrolled in the trial, 27,298 (83%) were followed and had valid anthropometric measurements at 6 months of age. We found no evidence of a difference in weight gain (mean difference 0.03 g/day, 95% confidence interval (CI) −0.12 to 0.18), height change (mean difference 0.004 mm/day, 95% CI −0.05 to 0.06), WAZ (mean difference −0.004 SD, 95% CI −0.03 to 0.02), WLZ (mean difference 0.001 SD, 95% CI −0.03 to 0.03), LAZ (mean difference −0.005 SD, 95% CI −0.03 to 0.02), or MUAC (mean difference 0.01 cm, 95% CI −0.01 to 0.04). The primary limitation of the trial was that measurements were only collected at enrollment and 6 months of age, precluding assessment of shorter-term or long-term changes in growth.

**Conclusions:**

Single-dose azithromycin does not appear to affect weight and height outcomes when administered during early infancy.

**Trial registration:**

ClinicalTrials.gov NCT03676764

## Abstract 2

Ali Sié and colleagues present pre-specified anthropometric outcomes from a randomized controlled trial that assessed the impact of a single-dose of oral azithromycin on infant mortality in Burkina Faso.

## Author summary

**Why was this study done?:**

- Antibiotics have been linked to obesity in children in nonrandomized studies in high-income settings.
- In low-income settings, malnutrition is a strong risk factor for child mortality, and interventions that increase weight gain may be beneficial.
- Single-dose azithromycin has previously been shown to reduce child mortality in sub-Saharan Africa, and it is possible that some of this effect is via weight gain.

**Why did the researchers do and find?:**

- We conducted a randomized controlled trial in which infants aged 5 to 12 weeks were randomly assigned to a single dose of azithromycin or a matching placebo.
- The primary outcome of the parent trial was all-cause mortality, and the trial found no evidence of a benefit of azithromycin for prevention of mortality.
- Infants were followed until 6 months of age and had anthropometric measurements, including height, weight, and mid-upper arm circumference, collected.
- We found no evidence of a difference in any anthropometric endpoint in infants randomized to azithromycin compared to placebo.

**What do these findings mean?:**

- Single-dose azithromycin does not appear to affect growth when administered to healthy infants who are 5 to 12 weeks of age.
- Growth outcomes were only measured at 6 months of age, and thus, any shorter or longer-term effects of azithromycin would not be detected by this study.
- This study only considered a single dose of azithromycin, as is used for prevention of child mortality and trachoma, and thus, it is possible that higher doses or longer duration of antibiotics could have a different effects on child growth.

## Introduction

Antibiotics are known to modulate the gut microbiome and are thought to be growth promoting in humans [1–5]. Some randomized controlled trials evaluating antibiotics in children with acute malnutrition, diarrhea, HIV, and other comorbidities have found increases in weight gain with children receiving antibiotics compared to placebo [6,7], while others have found no difference in growth outcomes [8]. In children who are HIV-exposed and uninfected, cotrimoxazole was associated with reduced risk of stunting [9]. Observational studies in high-income settings have suggested that early childhood antibiotic exposure is a risk factor for obesity and that there may be a dose-dependent response between antibiotic exposure and body mass index [10,11]. Most studies considering the effect of antibiotics on child growth have considered multiday antibiotic regimens, although any dose-dependent effect of antibiotics on growth is unclear.

In low- and middle-income countries, undernutrition contributes to approximately 45% of child deaths [12]. Underweight (low weight-for-age Z-score (WAZ)) is a strong predictor of mortality in children under 5 years of age and recently has been identified as one of the strongest anthropometric predictors of mortality among infants under 6 months of age [13–15]. Interventions to improve nutritional status in young infants must not interfere with exclusive breastfeeding, and thus, interventions such as supplemental or fortified foods are not appropriate in this age group. Mass drug administration with single-dose azithromycin has previously been shown to reduce all-cause childhood mortality among children 1 to 59 months of age in sub-Saharan Africa, with the largest effects in children aged 1 to 11 months in the highest mortality settings [16]. If antibiotics are growth promoting in the general population of infants, one potential mechanism of azithromycin for prevention of mortality may be via increased weight gain and reduced risk of underweight.

A previous study of single-dose azithromycin administered during the neonatal period for prevention of mortality found no overall effect of neonatal (age 8 to 27 days) azithromycin on 6-month mortality, but the study was underpowered due to a lower than anticipated mortality rate [17]. A single case of infantile hypertrophic pyloric stenosis was identified in that study in a male infant who received azithromycin [17]. In that trial, there was no evidence of an effect of single-dose azithromycin on infant anthropometric outcomes in the overall trial population [18]. These results were consistent with studies of growth among children under 5 years of age receiving single-dose azithromycin for trachoma control or prevention of mortality in cluster randomized trials [19–22]. In a subgroup analysis of the neonatal trial, neonates who were low birthweight (<2,500 g) and also underweight at enrollment (WAZ <−2 at age 8 to 27 days) had reduced odds of underweight at 6 months of age if they received azithromycin compared to placebo [23]. This trial excluded infants who were less than 2,500 g at enrollment (second to fourth weeks of life) for safety reasons, and thus, subgroups defined by the smallest infants were often underpowered. However, these results led to the hypothesis that single-dose azithromycin may not cause weight gain in the general population but may be growth promoting in subgroups of infants that are more vulnerable.

Here, we report data from a randomized controlled trial of over 30,000 infants in Burkina Faso of a single dose of azithromycin or placebo between the age of 5 and 12 weeks for prevention of infant mortality to evaluate effects of azithromycin on growth endpoints at 6 months of age. In the current trial, there were no enrollment exclusions based on weight, allowing for evaluation of a wider range of baseline anthropometric status. Based on the results of the neonatal trial [18], we hypothesized that overall, there would be no effect of azithromycin on growth endpoints but that there would be some benefit for infants with anthropometric deficits (e.g., underweight, stunting, and wasting) at enrollment.

## Methods

### Study setting

This study was conducted in the Nouna and Banfora districts of Burkina Faso. Burkina Faso is a landlocked country in West Africa in the Sahel that experiences seasonal rainfall from approximately July through October. Participants in the study were enrolled in Nouna District, in the northwest near the Mali border, and in Banfora, in the south.

### Trial methods

Complete methods for the trial have been previously reported [24]. In brief, the study was a 1:1 randomized placebo-controlled trial evaluating the efficacy of a single dose of azithromycin (20 mg/kg) or matching placebo for prevention of mortality by 6 months of age. Infants were enrolled from September 22, 2019, until October 22, 2022. The trial’s primary outcome, all-cause mortality, has been reported separately, as has adverse event data [25]. There was no evidence of a difference in the primary endpoint of the trial. This paper presents prespecified secondary endpoints, including weight gain (g/day), height change (mm/day), WAZ, weight-for-length Z-score (WLZ), and length-for-age Z-score (LAZ). The trial was reviewed and approved by the Institutional Review Board at the University of California, San Francisco, the Comite Institutionnel d’Ethique at the Centre de Reserche en Santé de Nouna, the Comite d’Ethique pour la Recherche en Santé in Ouagadougou, and the Comité Technique d’Examen des Demandes d’Autorisation d’Essais Cliniques in Ouagadougou. Written informed consent was obtained from the caregiver of each participant. The study was conducted in accordance with the Declaration of Helsinki.

The parent trial was designed to establish the efficacy of single-dose azithromycin administered during routine well-child visits (e.g., vaccination visits) during early infancy for prevention of all-cause mortality at 6 months of age. Infants were eligible for enrollment if they were between 5 and 12 weeks of age, were able to feed orally, their families were planning to stay in the study area for the full 6-month period of the study, and with appropriate caregiver consent. Infants were recruited directly in their community of residence via vaccine outreach teams that visit each community monthly, or in clinics during well-child visits [26]. During community outreach, mobilizers visited each study community to inform families that the study team was there, share some basic information about the study, and direct them to the study team if interested in participating. In clinics, families were informed of the study and were directed to the study team if interested in participation.

### Interventions

Participants were randomized in a 1:1 fashion without stratification or blocking to a single oral dose of azithromycin (20 mg/kg) or an equivalent volume of matching placebo. The placebo was identical to the azithromycin in taste, smell, and appearance. Study medications were donated by Pfizer (Pfizer, New York, NY). Participants were randomized and received their study medication after all baseline assessments, including anthropometric measurements, were completed. All study medication doses were directly observed.

### Anthropometric measurements

Anthropometric measurements were collected at baseline and at 6 months of age. Infants were weighed using a digital infant scale (ADE M112600U scale, Hamburg, Germany). Caregivers were instructed to remove bulky items of clothing prior to weighing each infant. The scale was standardized and calibrated each morning using a known test weight. Length was measured using a ShorrBoard (Weigh and Measure, Olney, MD). Due to the potential for measurement error, length measurements were taken in triplicate and the median was used for analysis. Mid-upper arm circumference (MUAC) was measured using a standard MUAC tape. WAZ (a marker of underweight), WLZ (a marker of wasting), and LAZ (a marker of stunting) were calculated using 2006 World Health Organization (WHO) reference standards [27]. Underweight was defined as WAZ <−2, wasting as WLZ <−2, and stunting as LAZ <−2. At 6 months of age, we additionally considered MUAC < 12.5 cm to be a sign of wasting.

### Outcomes

All prespecified secondary anthropometric endpoints at 6 months of age are included in this report, including weight gain (in g/day), height change (in mm/day), weight, length, WAZ, WLZ, LAZ, and MUAC. We additionally evaluated dichotomized post hoc outcomes of underweight, wasting defined by WLZ, wasting defined by MUAC, and stunting. Per our prespecified analysis plan, infants with values outside of WHO child growth standards for WAZ (−6 to +5 SD), LAZ (−6 to +6 SD), or WLZ (−5 to +5 SD) were excluded from the analysis. Additional endpoints included mortality, serious adverse events, hospitalizations, and clinic visits and are reported separately [25].

### Subgroup analyses

A series of non-prespecified subgroup analyses were conducted by the child’s age in months, sex, season of enrollment, at baseline underweight (WAZ < −2), wasting (WLZ < −2), and stunting (LAZ < −2) was assessed for all outcomes.

### Statistical methods

We used an intention-to-treat analysis for all outcomes. The prespecified analysis plan included superiority analyses for all outcomes. The randomization was not stratified, and the prespecified analysis plan did not include any adjustments for clustering. For weight gain (g/day) and height change (mm/day), we used an unadjusted linear regression model with a term for the infant’s randomized treatment assignment. For weight, length, WAZ, WLZ, LAZ, and MUAC, we used a linear regression model with a term for the randomized treatment assignment and adjusted for the baseline measure of each outcome variable. For underweight, wasting, and stunting outcomes, we used a logistic regression model with a single term for the infant’s randomized treatment assignment. For each subgroup for each outcome, a similar model was used as in the main analysis, with an interaction term between the subgroup and randomized treatment assignment to assess for the presence of effect modification on the additive scale. *P* < 0.05 was considered statistically significant, and all tests were two-sided. All analyses were run in R version 4.3.1 (The R Foundation for Statistical Computing, Vienna Austria).

## Results

**Fig 1:**
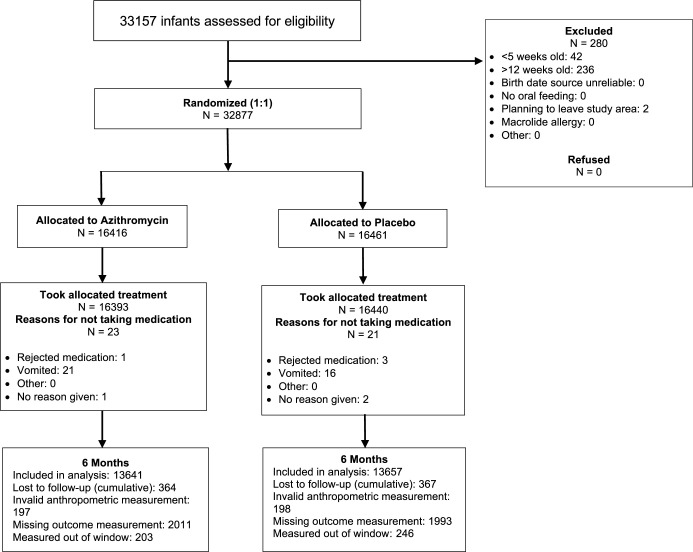
Screening, randomization, and follow-up of participants in a trial of azithromycin for infants aged 5–12 weeks of age.

Of 32,877 infants enrolled in the trial, 16,416 were randomized to azithromycin and 16,461 to placebo (**Fig 1**). Of these, 13,641 (83%) in the azithromycin arm and 13,657 (83%) in the placebo arm were included in the analysis. Reasons for being excluded from the analysis were that the infant had no anthropometric measurement (72%), the infant was lost to follow-up (13%), the infant was measured out of the prespecified visit window (8%), or the anthropometric measurement was not valid per WHO Child Growth Standards (7%; **Fig 1**). At baseline, infants were a median of 49 days old and 49% were female (**Table 1**). Baseline anthropometric measurements were similar between the 2 arms; median WLZ was −0.2 (standard deviation (SD), 1.5), median WAZ was −0.6 (SD 1.2), and median LAZ was −0.5 (SD 1.3) in each arm (**Table 1**). Baseline characteristics of infants who were and were not included in the analysis were similar (**Table A in S1 Appendix**).

**Table 1:** Baseline characteristics by randomized treatment group.

	Azithromycin (*N* = 16,416)	Placebo (*N* = 16,461)
**Age, days** Mean (SD)	48.9 (15.5)	49.3 (15.4)
**Sex, N (%)**		
Female	8,045 (49%)	8,136 (49%)
Male	8,371 (51%)	8,325 (51%)
**District, N (%)**		
Nouna	14,231 (87%)	14,271 (87%)
Banfora	1,298 (8%)	1,305 (8%)
Karankasso-Vigue	887 (5%)	885 (5%)
**Season of enrollment, N (%)**		
Rainy (June–October)	7,174 (44%)	7,279 (44%)
Dry (November–May)	9,242 (56%)	9,182 (56%)
**Weight at enrollment, kg**		
Mean (SD)	4.6 (0.9)	4.6 (0.9)
**Length at enrollment, cm**		
Mean (SD)	55.3 (3.0)	55.3 (3.1)
**WLZ**		
Mean (SD)	−0.2 (1.5)	−0.2 (1.5)
**WAZ**		
Mean (SD)	−0.6 (1.2)	−0.6 (1.2)
**LAZ**		
Mean (SD)	−0.5 (1.3)	−0.5 (1.3)
**MUAC**		
Median (IQR)	12.1 (1.2)	12.1 (1.2)

IQR, interquartile range; LAZ, length-for-age Z-score; MUAC, mid-upper arm circumference; SD, standard deviation; WAZ, weight-for-age Z-score; WLZ, weight-for-length Z-score.

At 6 months, we found no evidence of a difference in any anthropometric endpoint between the azithromycin and placebo arms in the overall population (**Table 2**). The mean value for each continuous anthropometric endpoint was nearly identical between the 2 arms. Similarly, we found no evidence of a difference in the prevalence of underweight, wasting, or stunting in infants receiving azithromycin compared to placebo at 6 months of age (**Table 3**).

**Table 2:** Anthropometric outcomes by randomized treatment group.

	Azithromycin Mean (SD)	Placebo Mean (SD)	Mean Difference (95% CI)	*P* value
N	13,641	13,657		
Age at follow-up, days^1^	185 (17)	185 (17)		
Weight gain, g/day	18.0 (6.2)	18.0 (6.4)	0.03 (−0.12 to 0.18)	0.67
Height change, mm/day	8.0 (2.1)	8.0 (2.3)	0.004 (−0.05 to 0.06)	0.87
Weight, kg^2^	7.1 (0.95)	7.1 (0.97)	0.005 (−0.01 to 0.02)	0.63
Length, cm^2^	66.1 (2.9)	66.1 (2.9)	0.01 (−0.05 to 0.08)	0.66
WAZ^2^	−0.74 (1.1)	−0.74 (1.1)	−0.004 (−0.03 to 0.02)	0.70
WLZ^2^	−0.64 (1.2)	−0.64 (1.2)	0.001 (−0.03 to 0.03)	0.94
LAZ^2^	−0.31 (1.3)	−0.31 (1.3)	−0.005 (−0.03 to 0.02)	0.73
MUAC, cm^2^	13.8 (1.2)	13.8 (1.2)	0.01 (−0.01 to 0.04)	0.37

CI, confidence interval; LAZ, length-for-age Z-score; MUAC, mid-upper arm circumference; SD, standard deviation; WAZ, weight-for-age Z-score; WLZ, weight-for-length Z-score.

^1^Infants were considered measured “in window” if they were between 120 and 270 days of age at the 6-month follow-up visit.

^2^Adjusted for baseline value of the outcome.

**Table 3:** Wasting, underweight, and stunting by randomized treatment group.

	Azithromycin N with outcome (%)	Placebo N with outcome (%)	Odds Ratio (95% CI)	*P* value
N	13,641	13,657		
Underweight (WAZ < −2)	1,656 (12.1%)	1,702 (12.5%)	0.97 (0.90 to 1.04)	0.42
Wasted (WLZ < −2)	1,701 (12.5%)	1,714 (12.6%)	0.99 (0.92 to 1.07)	0.84
Wasted (MUAC < 12.5 cm)	1,222 (9.0%)	1,265 (9.3%)	0.96 (0.89 to 1.05)	0.38
Stunted (LAZ < −2)	1,137 (8.3%)	1,149 (8.4%)	0.99 (0.91 to 1.08)	0.82

CI, confidence interval; LAZ, length-for-age Z-score; MUAC, mid-upper arm circumference; WAZ, weight-for-age Z-score; WLZ, weight-for-length Z-score.

**Fig 2:**
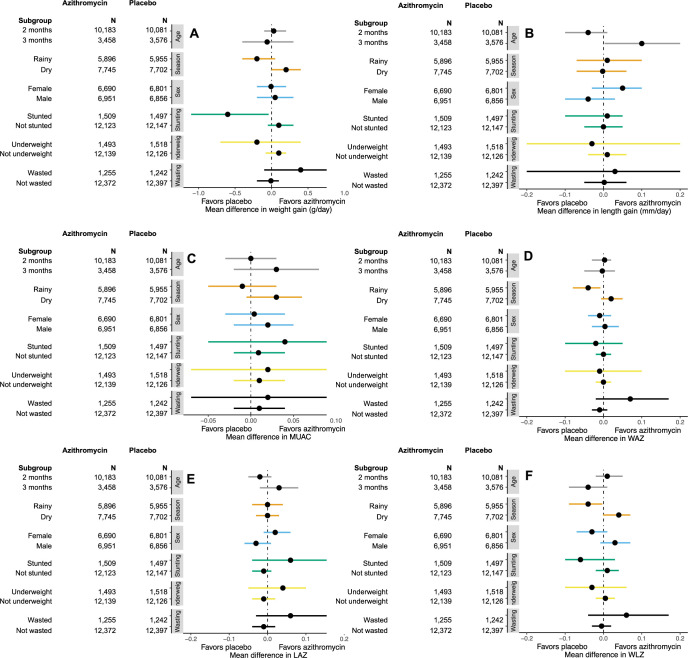
Differences in continuous anthropometric endpoints at 6 months of age in azithromycin compared to placebo among subgroups of infants defined by age at enrollment (grey bars), season of enrollment (orange bars), sex (blue bars), stunting (green bars), underweight (yellow bars), and wasting (black bars). Outcomes include weight gain in g/day (**A**), length change in mm/day (**B**), MUAC in cm (**C**), WAZ (**D**), LAZ (**E**), and WLZ (**F**). Means indicate mean value for the outcome in each subgroup by treatment arm, and N indicates the number of infants in each subgroup in each treatment arm. Mean differences for each outcome can be found in S1 Appendix. LAZ, length-for-age Z-score; MUAC, mid-upper arm circumference; WAZ, weight-for-age Z-score; WLZ, weight-for-length Z-score.

**Fig 3:**
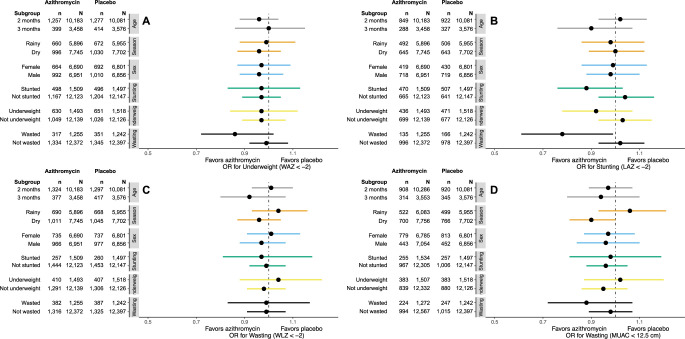
Differences in categorical anthropometric endpoints at 6 months of age in azithromycin compared to placebo among subgroups of infants defined by age at enrollment (grey bars), season of enrollment (orange bars), sex (blue bars), stunting (green bars), underweight (yellow bars), and wasting (black bars). Outcomes include underweight (WAZ, < −2; **A**), stunting (LAZ, < −2; **B**), wasting (WLZ, < −2; **C**), and wasting by MUAC (MUAC < 12.5 cm; **D**). For each outcome, n represented the number of infants with the outcome at 6 months in each randomized treatment arm, and N represents the total number of infants contributing to the subgroup. LAZ, length-for-age Z-score; MUAC, mid-upper arm circumference; WAZ, weight-for-age Z-score; WLZ, weight-for-length Z-score.

In a series of post hoc subgroup analyses, we found little consistent evidence of a difference in anthropometric endpoints in subgroups of children defined by age, sex, season, and baseline anthropometric status (**Figs 2** and **3 and Tables B-G in S1 Appendix**). Subgroup analyses by age at enrollment overall found little evidence of a difference in growth endpoints by age, but infants enrolled in their third month of life who were randomized to azithromycin versus placebo had greater gains in length between enrollment and 6 months compared to those enrolled in the second month (mean difference for second month: −0.04 mm/day, 95% confidence interval (CI) −0.1 to 0.01; mean difference for second month: 0.1 mm/day, 95% CI 0.003 to 0.2, P for interaction = 0.008; **Table B in S1 Appendix**). Infants who were wasted at baseline (WLZ < −2) who received azithromycin had reduced odds of stunting at 6 months (OR 0.78, 95% CI 0.61 to 0.99, *P* for interaction = 0.04) compared to those who received placebo (**Table F in S1 Appendix**). Infants who were stunted at baseline who received azithromycin had reduced weight gain compared to those who received placebo (mean difference −0.6 g/day, 95% CI −1.1 to −0.04, *P* for interaction = 0.006; **Table G in S1 Appendix**).

## Discussion

Overall, we found no evidence of a difference in growth outcomes at 6 months of age in infants receiving a single oral dose of azithromycin compared to placebo. These results accord with previous results of azithromycin administration during the neonatal period that found no evidence of an effect of single-dose azithromycin on 6-month anthropometric measurements in Burkina Faso [18]. Similarly, studies of mass distribution of azithromycin for prevention of mortality and for trachoma control have generally not found evidence of a growth benefit from single-dose azithromycin among children under 5 years of age [19–22]. Taken together, these results suggest that in the general population, single-dose azithromycin administered during infancy does not improve growth outcomes in the months following administration.

The current trial collected anthropometric measurements at baseline (5 to 12 weeks of age) and at 6 months of age, limiting ability to make inferences about shorter-term or longer-term changes in anthropometric outcomes. However, infants were enrolled between 5 and 12 weeks of age, meaning that the 6-month endpoint ranged between 3 and 5 months after treatment for most infants. In subgroup analyses by age in months, there was little evidence of a difference in growth endpoints by age, although infants in the third month of life had greater height change with azithromycin compared to placebo versus those in the second month of life, although absolute differences were small. It is possible that azithromycin has some short-term effects that lead to short-term increases in height that do not persist or that there are benefits in treating older infants that are not observed in younger infants. However, multiple subgroup analyses were conducted, and thus, this finding may also be due to chance.

Randomized controlled trials of antibiotics for children with severe acute malnutrition have found a growth-promoting effect of antibiotics in the short term when antibiotics are included as part of a treatment program for uncomplicated severe acute malnutrition compared to amoxicillin [7,28]. However, these differences were typically only apparent in the weeks following treatment, with no evidence of a difference in weight gain several months after treatment administration [29]. Currently, most treatment guidelines for children with undernutrition are focused on children over 6 months of age. Thus, the role of antibiotics in infants <6 months of age who are undernourished is unclear. In subgroup analyses, we were unable to demonstrate a difference in anthropometric outcomes following azithromycin administration in infants who were underweight (WAZ < −2) at baseline. We found contradictory results between infants with wasting and stunting at baseline, with infants with wasting randomized to azithromycin having reduced odds of stunting, but infants with stunting randomized to azithromycin gaining less weight. These results do not clearly indicate a benefit of early infancy antibiotics for children with undernutrition.

A major concern related to early life antibiotic administration has been the risk of weight gain and obesity in exposed children in high-income settings [3–5]. Antibiotics have been shown to be growth promoting in livestock [30,31]. This mechanism may be via treatment of infection or alteration of the gut microbiome. In the present analysis, while we did not show evidence of a difference in weight gain in infants receiving azithromycin compared to placebo, we are unable to comment on obesity, as the prevalence of overweight and obesity was too low in this population for meaningful inferences and infants were only followed until 6 months of age. Weight gain among infants in rural Burkina Faso is typically considered to be beneficial, and thus, any improvement in weight gain could be viewed as health promoting in this population. While the absence of increases in weight gain following azithromycin compared to placebo suggests it is unlikely that single-dose azithromycin causes obesity among infants, it is unclear if these results would generalize to populations with a higher prevalence of overweight among infants.

This study has several limitations. First, although the overall sample size was large, the sample size for some subgroups was limited. In particular, we did not have statistical power to evaluate subgroups with severe underweight, wasting, or stunting (WAZ, WLZ, and LAZ < −3, respectively) or concurrent wasting and stunting. Infants in these categories have the highest risk of mortality, morbidity, and poor developmental outcomes and are a priority population for intervention. We are unable to comment on the role of single-dose azithromycin in these groups of children. Second, multiple subgroup analyses were conducted, and thus, it is possible that statistically significant findings were due to chance. Most differences were small and not clinically meaningful. The results of the subgroup analyses should be treated as hypothesis generating. Third, we collected anthropometric measurements only at enrollment (5 to 12 weeks of age) and 6 months of age. Any more proximal or long-term effects of azithromycin on anthropometric endpoints in infants would not be detected in these analyses. However, it is unlikely that a long-term difference in growth would be observed given the lack of effect at 6 months, and the clinical significance of any shorter-term changes is unclear given the lack of effect at 6 months. Fourth, this study evaluated a single 20 mg/kg dose of azithromycin compared to placebo, which is the dosing currently being considered for programmatic scale-up for prevention of child mortality and is the dosing used by trachoma programs for children [32,33]. Higher doses or repeated exposure to antibiotics may result in different outcomes, and we cannot comment on the effect of different antibiotics or different dosing regimens. Previous work in the study area has suggested that infants receive several courses of antibiotics per year [34,35]. Future studies considering the impact of repeated courses of antibiotics may yield additional insights. Finally, these results are likely only generalizable to populations of infants with a similar distribution of weight and height and with similar dietary and disease patterns.

Overall, we found no evidence of an effect of infant administration of a single dose of oral azithromycin compared to placebo on weight- or height-based outcomes. These results confirm earlier work evaluating single-dose azithromycin administered during the neonatal period [18]. Taken together, the 2 trials, which, in total, enrolled over 50,000 infants, provide definitive evidence that single-dose azithromycin does not cause weight or height changes in infants in Burkina Faso.

## Supporting information

S1 Appendix Supporting data tables. **Table A.** Baseline characteristics among infants included in the analysis (*N* = 27,743) and not included in the analysis (*N* = 5,134) by randomized treatment assignment. **Table B.** Results of subgroup analyses for each outcome at 6 months of age by age at enrollment in months. **Table C.** Results of subgroup analyses for each outcome at 6 months of age by child’s sex. **Table D.** Results of subgroup analyses for each outcome at 6 months of age by season of enrollment. **Table E.** Results of subgroup analyses for each outcome at 6 months of age by underweight (weight-for-age Z-score <− 2) at enrollment. **Table F.** Results of subgroup analyses for each outcome at 6 months of age by wasting (weight-for-length Z-score <− 2) at enrollment. **Table G.** Results of subgroup analyses for each outcome at 6 months of age by stunting (height-for-length Z-score <− 2) at enrollment
(DOCX)

S2 Appendix Statistical analysis plan for the trial. (PDF)

## Abbreviations

CI: confidence interval
LAZ: length-for-age Z-score
MUAC: mid-upper arm circumference
SD: standard deviation
WAZ: weight-for-age Z-score
WHO: World Health Organization
WLZ: weight-for-length Z-score
