# Supplementary material for: Single-dose azithromycin for infant growth in Burkina Faso: Prespecified secondary anthropometric outcomes from a randomized controlled trial
Source: PLoS Med. 2024 Jan 23;21(1):e1004345. doi: 10.1371/journal.pmed.1004345 (PMC10846702; doi:10.1371/journal.pmed.1004345)
Supplement: S1 Appendix — Table A. Baseline characteristics among infants included in the analysis (N = 27,743) and not included in the analysis (N = 5,134) by randomized treatment assignment. Table B. Results of subgroup analyses for each outcome at 6 months of age by age at enrollment in months. Table C. Results of subgroup analyses for each outcome at 6 months of age by child’s sex. Table D. Results of subgroup analyses for each outcome at 6 months of age by season of enrollment. Table E. Results of subgroup analyses for each outcome at 6 months of age by underweight (weight-for-age Z-score <− 2) at enrollment. Table F. Results of subgroup analyses for each outcome at 6 months of age by wasting (weight-for-length Z-score <− 2) at enrollment. Table G. Results of subgroup analyses for each outcome at 6 months of age by stunting (height-for-length Z-score <− 2) at enrollment (DOCX) [file pmed.1004345.s001.docx]

**Supplemental Appendix S1**

Supplement to: Sié A, Ouattara M, Bountogo M, Dah C, et al. Single dose azithromycin for infant growth in Burkina Faso: prespecified secondary anthropometric outcomes from a randomized controlled trial

**Table A.** Baseline characteristics among infants included in the analysis (N=27,743) and not included in the analysis (N=5,134) by randomized treatment assignment

|  | **Included in Analysis** | | **Not Included in Analysis^1^** | |
| --- | --- | --- | --- | --- |
|  | *Azithromycin (N=13,641)* | *Placebo*  *(N=13,657)* | *Azithromycin (N=2,775)* | *Placebo*  *(N=2,804)* |
| **Age, days**  Mean (SD) | 48.5 (15.4) | 49.0 (15.3) | 51.2 (15.8) | 51.2 (15.7) |
| **Sex, N (%)** |  |  |  |  |
| Female | 6,690 (49%) | 6,801 (50%) | 1,355 (49%) | 1,335 (48%) |
| Male | 6,951 (51%) | 6,856 (50%) | 1,420 (51%) | 1,469 (52%) |
| **District, N (%)** |  |  |  |  |
| Nouna | 11,917 (87%) | 12,020 (88%) | 2,314 (83%) | 2,251 (80%) |
| Banfora | 1,080 (8%) | 1,047 (8%) | 218 (8%) | 258 (9%) |
| Karankasso-Vigue | 644 (5%) | 590 (4%) | 243 (9%) | 295 (11%) |
| **Season of enrollment, N (%)** |  |  |  |  |
| Rainy (June-October) | 5,896 (43%) | 5,955 (44%) | 1,278 (46%) | 1,324 (47%) |
| Dry (November-May) | 7,745 (57%) | 7,702 (56%) | 1,497 (54%) | 1,480 (53%) |
| **Weight at enrollment, kg** |  |  |  |  |
| Mean (SD) | 4.6 (0.8) | 4.6 (0.9) | 4.7 (1.0) | 4.7 (1.0) |
| **Length at enrollment, cm** |  |  |  |  |
| Mean (SD) | 55.3 (3.0) | 55.3 (3.0) | 55.5 (3.4) | 55.4 (3.5) |
| **WLZ** |  |  |  |  |
| Mean (SD) | -0.2 (1.4) | -0.2 (1.4) | -0.3 (1.9) | -0.2 (1.9) |
| **WAZ** |  |  |  |  |
| Mean (SD) | -0.6 (1.1) | -0.6 (1.2) | -0.7 (1.4) | -0.7 (1.4) |
| **LAZ** |  |  |  |  |
| Mean (SD) | -0.5 (1.2) | -0.5 (1.2) | -0.5 (1.5) | -0.6 (1.5) |
| **MUAC** |  |  |  |  |
| Median (IQR) | 12.1 (1.2) | 12.1 (1.2) | 12.1 (1.3) | 12.1 (1.3) |

^1^Infants were not included in the analysis if they were lost to follow-up at 6 months, had missing outcome measurements, had measurements outside of the pre-specified follow-up window, or had measurements outside of the World Health Organization child growth standards.

**Table B.** Results of subgroup analyses for each outcome at 6 months of age by age at enrollment in months

|  | **Azithromycin** | | | **Placebo** | | |  | |
| --- | --- | --- | --- | --- | --- | --- | --- | --- |
| **Outcome** | **N** | **Mean (SD)** | | **N** | **Mean (SD)** | | **Mean Difference**  **(95 % CI)** | **P-value^1^** |
| **Weight gain (g/day)** | | | |  | | | | |
| 2 months | 10,183 | 18.8 (5.9) | | 10,081 | 18.7 (6.1) | | 0.03 (-0.1 to 0.2) | 0.62 |
| 3 months | 3,458 | 15.8 (6.6) | | 3,576 | 15.8 (6.6) | | -0.06 (-0.4 to 0.3) |  |
| **Height change (mm/day)** | | | |  | | | | |
| 2 months | 10,183 | 8.1 (2.0) | | 10,081 | 8.2 (2.1) | | -0.04 (-0.1 to 0.01) | 0.008 |
| 3 months | 3,458 | 7.5 (2.5) | | 3,576 | 7.3 (2.4) | | 0.1 (0.003 to 0.2) |  |
| **WAZ** |  |  |  |  |  |  |  |  |
| 2 months | 10,183 | -0.8 (1.1) | | 10,081 | -0.8 (1.1) | | 0.003 (-0.03 to 0.02) | 0.85 |
| 3 months | 3,458 | -0.7 (1.1) | | 3,576 | -0.7 (1.2) | | -0.003 (-0.05 to 0.03) |  |
| **WLZ** |  |  |  |  |  |  |  |  |
| 2 months | 10,183 | -0.7 (1.2) | | 10,081 | -0.7 (1.2) | | 0.01 (-0.02 to 0.05) | 0.12 |
| 3 months | 3,458 | -0.5 (1.2) | | 3,576 | -0.5 (1.2) | | -0.04 (-0.09 to 0.01) |  |
| **MUAC (cm)** |  |  | |  |  | |  |  |
| 2 months | 10,183 | 13.8 (1.2) | | 10,081 | 13.8 (1.2) | | 0.00 (-0.03 to 0.03) | 0.25 |
| 3 months | 3,458 | 13.8 (1.2) | | 3,576 | 13.8 (1.2) | | 0.03 (-0.01 to 0.08) |  |
| **LAZ** |  |  | |  |  | |  |  |
| 2 months | 10,183 | -0.3 (1.3) | | 10,081 | -0.3 (1.3) | | -0.02 (-0.05 to 0.01) | 0.12 |
| 3 months | 3,458 | -0.4 (1.2) | | 3,576 | -0.4 (1.3) | | 0.03 (-0.02 to 0.08) |  |
|  | **Azithromycin** | | | **Placebo** |  | |  |  |
|  | **N with outcome** | | | **N with outcome** | | | **Odds Ratio**  **(95 % CI)** | **P-value^1^** |
| **Underweight^2^** |  | | |  | | |  |  |
| 2 months | 1,257 (12.3%) | | | 1,277 (12.8%) | | | 0.96 (0.88 to 1.04) | 0.68 |
| 3 months | 399 (11.5%) | | | 414 (11.6%) | | | 1.00 (0.86 to 1.15) |  |
| **Wasted (WLZ)^3^** |  |  | |  |  | |  |  |
| 2 months | 1,324 (13.0%) | | | 1,297 (12.9%) | | | 1.01 (0.93 to 1.10) | 0.31 |
| 3 months | 377 (10.9%) | | | 417 (11.7%) | | | 0.93 (0.80 to 1.07) |  |
| **Wasted (MUAC)^4^** |  |  | |  |  | |  |  |
| 2 months | 908 (8.9%) | | | 920 (9.1%) | | | 0.97 (0.89 to 1.07) | 0.67 |
| 3 months | 314 (9.1%) | | | 345 (9.7%) | | | 0.94 (0.80 to 1.10) |  |
| **Stunted^5^** |  |  | |  |  | |  |  |
| 2 months | 849 (8.3%) | | | 922 (8.3%) | | | 1.02 (0.93 to 1.13) | 0.20 |
| 3 months | 288 (8.3%) | | | 327 (9.1%) | | | 0.90 (0.76 to 1.07) |  |

Abbreviations: SD: standard deviation, CI: confidence interval, g, grams, mm, millimeters, WAZ, weight-for-age Z-score, WLZ: weight-for-length Z-score, MUAC: mid-upper arm circumference; ^1^P for interaction for subgroup category by randomized treatment assignment; ^2^Defined as WAZ < -2; ^3^Defined as WLZ < -2; ^4^Defined as MUAC < 12.5 cm; ^5^Defined as LAZ < -2.

**Table C.** Results of subgroup analyses for each outcome at 6 months of age by child’s sex

|  | **Azithromycin** | | | **Placebo** | | |  | |
| --- | --- | --- | --- | --- | --- | --- | --- | --- |
| **Outcome** | **N** | **Mean (SD)** | | **N** | **Mean (SD)** | | **Mean Difference**  **(95 % CI)** | **P-value^1^** |
| **Weight gain (g/day)** | | | |  | | | | |
| Female | 6,690 | 17.4 (6.0) | | 6,801 | 17.4 (6.3) | | -0.007 (-0.2 to 0.2) | 0.69 |
| Male | 6,951 | 18.6 (6.3) | | 6,856 | 18.5 (6.4) | | 0.05 (-0.2 to 0.3) |  |
| **Height change (mm/day)** | | | |  | | | | |
| Female | 6,690 | 7.9 (2.1) | | 6,801 | 7.8 (2.4) | | 0.05 (-0.03 to 0.1) | 0.11 |
| Male | 6,951 | 8.1 (2.1) | | 6,856 | 8.1 (2.1) | | -0.04 (-0.1 to 0.03) |  |
| **WAZ** |  |  |  |  |  |  |  |  |
| Female | 6,690 | -0.6 (1.1) | | 6,801 | -0.6 (1.1) | | -0.01 (-0.04 to 0.02) | 0.51 |
| Male | 6,951 | -0.8 (1.2) | | 6,856 | -0.8 (1.3) | | 0.004 (-0.03 to 0.04) |  |
| **WLZ** |  |  |  |  |  |  |  |  |
| Female | 6,690 | -0.6 (1.2) | | 6,801 | -0.6 (1.2) | | -0.03 (-0.07 to 0.01) | 0.03 |
| Male | 6,951 | -0.7 (1.3) | | 6,856 | -0.7 (1.3) | | 0.03 (-0.008 to 0.07) |  |
| **MUAC (cm)** |  |  | |  |  | |  |  |
| Female | 6,690 | 13.6 (1.2) | | 6,801 | 13.6 (1.2) | | 0.004 (-0.03 to 0.04) | 0.65 |
| Male | 6,951 | 14.0 (1.2) | | 6,856 | 14.0 (1.2) | | 0.02 (-0.02 to 0.05) |  |
| **LAZ** |  |  | |  |  | |  |  |
| Female | 6,690 | -0.1 (1.2) | | 6,801 | -0.2 (1.2) | | 0.02 (-0.01 to 0.06) | 0.06 |
| Male | 6,951 | -0.5 (1.3) | | 6,856 | -0.5 (1.3) | | -0.03 (-0.06 to 0.009) |  |
|  | **Azithromycin** | | | **Placebo** |  | |  |  |
|  | **N with outcome** | | | **N with outcome** | | | **Odds Ratio**  **(95 % CI)** | **P-value^1^** |
| **Underweight^2^** |  | | |  | | |  |  |
| Female | 664 (9.9%) | | | 692 (10.2%) | | | 0.97 (0.87 to 1.09) | 0.90 |
| Male | 992 (14.3%) | | | 1,010 (14.7%) | | | 0.96 (0.88 to 1.06) |  |
| **Wasted (WLZ)^3^** |  |  | |  |  | |  |  |
| Female | 735 (11.0%) | | | 737 (10.8%) | | | 1.02 (0.91 to 1.13) | 0.55 |
| Male | 966 (13.9%) | | | 977 (14.3%) | | | 0.97 (0.88 to 1.07) |  |
| **Wasted (MUAC)^4^** |  |  | |  |  | |  |  |
| Female | 779 (11.6%) | | | 813 (12.0%) | | | 0.97 (0.87 to 1.08) | 0.94 |
| Male | 443 (6.4%) | | | 452 (6.6%) | | | 0.96 (0.84 to 1.10) |  |
| **Stunted^5^** |  |  | |  |  | |  |  |
| Female | 419 (6.3%) | | | 430 (6.3%) | | | 0.99 (0.86 to 1.13) | 0.94 |
| Male | 718 (10.3%) | | | 719 (10.5%) | | | 0.98 (0.88 to 1.10) |  |

Abbreviations: SD: standard deviation, CI: confidence interval, g, grams, mm, millimeters, WAZ, weight-for-age Z-score, WLZ: weight-for-length Z-score, MUAC: mid-upper arm circumference; ^1^P for interaction for subgroup category by randomized treatment assignment; ^2^Defined as WAZ < -2; ^3^Defined as WLZ < -2; ^4^Defined as MUAC < 12.5 cm; ^5^Defined as LAZ < -2.

**Table D.** Results of subgroup analyses for each outcome at 6 months of age by season of enrollment

|  | **Azithromycin** | | | **Placebo** | | |  | |
| --- | --- | --- | --- | --- | --- | --- | --- | --- |
| **Outcome** | **N** | **Mean (SD)** | | **N** | **Mean (SD)** | | **Mean Difference**  **(95 % CI)** | **P-value^1^** |
| **Weight gain (g/day)** | | | |  | | | | |
| Rainy | 5,896 | 18.0 (6.1) | | 5,955 | 18.2 (6.6) | | -0.2 (-0.4 to 0.05) | 0.01 |
| Dry | 7,745 | 18.0 (6.3) | | 7,702 | 17.8 (6.2) | | 0.2 (0.0 to 0.4) |  |
| **Height change (mm/day)** | | | |  | | | | |
| Rainy | 5,896 | 8.0 (2.1) | | 5,955 | 8.0 (2.5) | | 0.01 (-0.07 to 0.10) | 0.78 |
| Dry | 7,745 | 8.0 (2.1) | | 7,702 | 8.0 (2.1) | | -0.002 (-0.07 to 0.06) |  |
| **WAZ** |  |  |  |  |  |  |  |  |
| Rainy | 5,896 | -0.7 (1.1) | | 5,955 | -0.7 (1.1) | | -0.04 (-0.08 to -0.008) | 0.004 |
| Dry | 7,745 | -0.8 (1.1) | | 7,702 | -0.8 (1.1) | | 0.02 (-0.006 to 0.05) |  |
| **WLZ** |  |  |  |  |  |  |  |  |
| Rainy | 5,896 | -0.6 (1.2) | | 5,955 | -0.6 (1.2) | | -0.04 (-0.09 to -0.003) | 0.005 |
| Dry | 7,745 | -0.7 (1.2) | | 7,702 | -0.7(1.3) | | 0.04 (-0.001 to 0.07) |  |
| **MUAC (cm)** |  |  | |  |  | |  |  |
| Rainy | 5,896 | 13.8 (1.2) | | 5,955 | 13.9 (1.2) | | -0.01 (-0.05 to 0.03) | 0.14 |
| Dry | 7,745 | 13.8 (1.2) | | 7,702 | 13.8 (1.2) | | 0.03 (-0.005 to 0.06) |  |
| **LAZ** |  |  | |  |  | |  |  |
| Rainy | 5,896 | -0.3 (1.3) | | 5,955 | -0.3 (1.3) | | 0.00 (-0.04 to 0.04) | 0.70 |
| Dry | 7,745 | -0.3 (1.2) | | 7,702 | -0.3 (1.3) | | 0.00 (-0.03 to 0.03) |  |
|  | **Azithromycin** | | | **Placebo** |  | |  |  |
|  | **N with outcome** | | | **N with outcome** | | | **Odds Ratio**  **(95 % CI)** | **P-value^1^** |
| **Underweight^2^** |  | | |  | | |  |  |
| Rainy | 660 (11.2%) | | | 672 (11.3%) | | | 0.99 (0.88 to 1.11) | 0.63 |
| Dry | 996 (12.9%) | | | 1,030 (13.4%) | | | 0.96 (0.87 to 1.05) |  |
| **Wasted (WLZ)^3^** |  |  | |  |  | |  |  |
| Rainy | 690 (11.7%) | | | 668 (11.2%) | | | 1.05 (0.94 to 1.17) | 0.21 |
| Dry | 1,011 (13.1%) | | | 1,045 (13.6%) | | | 0.96 (0.87 to 1.05) |  |
| **Wasted (MUAC)^4^** |  |  | |  |  | |  |  |
| Rainy | 522 (8.9%) | | | 499 (8.4%) | | | 1.06 (0.93 to 1.21) | 0.05 |
| Dry | 700 (9.0%) | | | 766 (10.0%) | | | 0.90 (0.81 to 1.00) |  |
| **Stunted^5^** |  |  | |  |  | |  |  |
| Rainy | 492 (8.3%) | | | 506 (8.5%) | | | 0.98 (0.86 to 1.12) | 0.85 |
| Dry | 645 (8.3%) | | | 643 (8.4%) | | | 1.00 (0.89 to 1.12) |  |

Abbreviations: SD: standard deviation, CI: confidence interval, g, grams, mm, millimeters, WAZ, weight-for-age Z-score, WLZ: weight-for-length Z-score, MUAC: mid-upper arm circumference; ^1^P for interaction for subgroup category by randomized treatment assignment; ^2^Defined as WAZ < -2; ^3^Defined as WLZ < -2; ^4^Defined as MUAC < 12.5 cm; ^5^Defined as LAZ < -2

**Table E.** Results of subgroup analyses for each outcome at 6 months of age by underweight (weight-for-age Z-score < - 2) at enrollment

|  | **Azithromycin** | | | **Placebo** | | |  | |
| --- | --- | --- | --- | --- | --- | --- | --- | --- |
| **Outcome** | **N** | **Mean (SD)** | | **N** | **Mean (SD)** | | **Mean Difference**  **(95 % CI)** | **P-value^1^** |
| **Weight gain (g/day)** | | | |  | | | | |
| Underweight | 1,507 | 21.3 (7.3) | | 1,547 | 21.5 (8.3) | | -0.2 (-0.7 to 0.4) | 0.30 |
| Not underweight | 12,332 | 17.6 (6.0) | | 12,357 | 17.5 (5.9) | | 0.1 (-0.08 to 0.2) |  |
| **Height change (mm/day)** | | | |  | | | | |
| Underweight | 1,493 | 8.7 (2.3) | | 1,518 | 8.7 (3.4) | | -0.03 (-0.2 to 0.2) | 0.62 |
| Not underweight | 12,139 | 7.9 (2.0) | | 12,126 | 7.9 (2.0) | | 0.01 (-0.04 to 0.06) |  |
| **WAZ** |  |  |  |  |  |  |  |  |
| Underweight | 1,493 | -1.8 (1.3) | | 1,518 | -1.8 (1.3) | | -0.01 (-0.1 to 0.1) | 0.60 |
| Not underweight | 12,139 | -0.6 (1.0) | | 12,126 | -0.6 (1.1) | | 0.00 (-0.02 to 0.02) |  |
| **WLZ** |  |  |  |  |  |  |  |  |
| Underweight | 1,493 | -1.3 (1.3) | | 1,518 | -1.2 (1.3) | | -0.03 (-0.1 to 0.06) | 0.37 |
| Not underweight | 12,139 | -0.6 (1.2) | | 12,126 | -0.6 (1.2) | | 0.005 (-0.02 to 0.03) |  |
| **MUAC (cm)** |  |  | |  |  | |  |  |
| Underweight | 1,493 | 13.2 (1.3) | | 1,518 | 13.1 (1.3) | | 0.02 (-0.07 to 0.1) | 0.85 |
| Not underweight | 12,139 | 13.9 (1.1) | | 12,126 | 13.9 (1.2) | | 0.01 (-0.02 to 0.04) |  |
| **LAZ** |  |  | |  |  | |  |  |
| Underweight | 1,493 | -1.3 (1.4) | | 1,518 | -1.3 (1.4) | | 0.04 (-0.05 to 0.1) | 0.21 |
| Not underweight | 12,139 | -0.2 (1.2) | | 12,126 | -0.2 (1.2) | | -0.01 (-0.04 to 0.02) |  |
|  | **Azithromycin** | | | **Placebo** |  | |  |  |
|  | **N with outcome** | | | **N with outcome** | | | **Odds Ratio**  **(95 % CI)** | **P-value^1^** |
| **Underweight^2^** |  | | |  | | |  |  |
| Underweight | 630 (42.2%) | | | 651 (42.9%) | | | 0.97 (0.84 to 1.12) | 0.98 |
| Not underweight | 1,049 (8.7%) | | | 1,026 (8.5%) | | | 0.97 (0.89 to 1.07) |  |
| **Wasted (WLZ)^3^** |  |  | |  |  | |  |  |
| Underweight | 410 (27.5%) | | | 407 (26.8%) | | | 1.03 (0.88 to 1.21) | 0.61 |
| Not underweight | 1,291 (10.6%) | | | 1,306 (10.8%) | | | 0.99 (0.91 to 1.07) |  |
| **Wasted (MUAC)^4^** |  |  | |  |  | |  |  |
| Underweight | 383 (25.7%) | | | 383 (25.2%) | | | 1.02 (0.87 to 1.20) | 0.44 |
| Not underweight | 839 (6.9%) | | | 880 (7.3%) | | | 0.95 (0.86 to 1.05) |  |
| **Stunted^5^** |  |  | |  |  | |  |  |
| Underweight | 436 (29.2%) | | | 471 (31.0%) | | | 0.92 (0.78 to 1.07) | 0.22 |
| Not underweight | 699 (5.8%) | | | 677 (5.6%) | | | 1.03 (0.93 to 1.15) |  |

Abbreviations: SD: standard deviation, CI: confidence interval, g, grams, mm, millimeters, WAZ, weight-for-age Z-score, WLZ: weight-for-length Z-score, MUAC: mid-upper arm circumference; ^1^P for interaction for subgroup category by randomized treatment assignment; ^2^Defined as WAZ < -2; ^3^Defined as WLZ < -2; ^4^Defined as MUAC < 12.5 cm; ^5^Defined as LAZ < -2.

**Table F.** Results of subgroup analyses for each outcome at 6 months of age by wasting (weight-for-length Z-score < - 2) at enrollment

|  | **Azithromycin** | | | **Placebo** | | |  | |
| --- | --- | --- | --- | --- | --- | --- | --- | --- |
| **Outcome** | **N** | **Mean (SD)** | | **N** | **Mean (SD)** | | **Mean Difference**  **(95 % CI)** | **P-value^1^** |
| **Weight gain (g/day)** | | | |  | | | | |
| Wasted | 1,255 | 21.0 (7.2) | | 1,242 | 20.6 (7.2) | | 0.4 (-0.1 to 1.0) | 0.08 |
| Not wasted | 12,372 | 17.7 (6.0) | | 12,397 | 17.7 (6.2) | | -0.008 (-0.2 to 0.1) |  |
| **Height change (mm/day)** | | | |  | | | | |
| Wasted | 1,255 | 7.4 (2.4) | | 1,242 | 7.3 (2.3) | | 0.03 (-0.2 to 0.2) | 0.73 |
| Not wasted | 12,372 | 8.0 (2.1) | | 12,397 | 8.0 (2.2) | | 0.002 (-0.05 to 0.06) |  |
| **WAZ** |  |  |  |  |  |  |  |  |
| Wasted | 1,255 | -1.2 (1.3) | | 1,242 | -1.4 (1.3) | | 0.07 (-0.02 to 0.17) | 0.11 |
| Not wasted | 12,372 | -0.7 (1.1) | | 12,397 | -0.7 (1.1) | | -0.01 (-0.03 to 0.01) |  |
| **WLZ** |  |  |  |  |  |  |  |  |
| Wasted | 1,255 | -1.3 (1.3) | | 1,242 | -1.4 (1.4) | | 0.06 (-0.04 to 0.17) | 0.17 |
| Not wasted | 12,372 | -0.6 (1.2) | | 12,397 | -0.6 (1.2) | | -0.005 (-0.03 to 0.02) |  |
| **MUAC (cm)** |  |  | |  |  | |  |  |
| Wasted | 1,255 | 13.4 (1.3) | | 1,242 | 13.4 (1.3) | | 0.02 (-0.07 to 0.11) | 0.83 |
| Not wasted | 12,372 | 13.9 (1.2) | | 12,397 | 13.9 (1.2) | | 0.01 (-0.02 to 0.04) |  |
| **LAZ** |  |  | |  |  | |  |  |
| Wasted | 1,255 | -0.2 (1.4) | | 1,242 | -0.4 (1.5) | | 0.06 (-0.03 to 0.16) | 0.12 |
| Not wasted | 12,372 | -0.3 (1.2) | | 12,397 | -0.3 (1.2) | | -0.01 (-0.04 to 0.02) |  |
|  | **Azithromycin** | | | **Placebo** |  | |  |  |
|  | **N with outcome** | | | **N with outcome** | | | **Odds Ratio**  **(95 % CI)** | **P-value^1^** |
| **Underweight^2^** |  | | |  | | |  |  |
| Wasted | 317 (25.3%) | | | 351 (28.3%) | | | 0.86 (0.72 to 1.02) | 0.14 |
| Not wasted | 1,334 (10.8%) | | | 1,345 (10.9%) | | | 0.99 (0.92 to 1.08) |  |
| **Wasted (WLZ)^3^** |  |  | |  |  | |  |  |
| Wasted | 382 (30.4%) | | | 387 (31.2%) | | | 0.97 (0.82 to 1.15) | 0.77 |
| Not wasted | 1,316 (10.6%) | | | 1,325 (10.7%) | | | 0.99 (0.92 to 1.08) |  |
| **Wasted (MUAC)^4^** |  |  | |  |  | |  |  |
| Wasted | 224 (17.9%) | | | 247 (19.9%) | | | 0.88 (0.72 to 1.07) | 0.32 |
| Not wasted | 994 (8.0%) | | | 1,015 (8.2%) | | | 0.98 (0.89 to 1.07) |  |
| **Stunted^5^** |  |  | |  |  | |  |  |
| Wasted | 135 (10.8%) | | | 166 (13.4%) | | | 0.78 (0.61 to 0.99) | 0.04 |
| Not wasted | 996 (8.1%) | | | 978 (7.9%) | | | 1.02 (0.93 to 1.12) |  |

Abbreviations: SD: standard deviation, CI: confidence interval, g, grams, mm, millimeters, WAZ, weight-for-age Z-score, WLZ: weight-for-length Z-score, MUAC: mid-upper arm circumference; ^1^P for interaction for subgroup category by randomized treatment assignment; ^2^Defined as WAZ < -2; ^3^Defined as WLZ < -2; ^4^Defined as MUAC < 12.5 cm; ^5^Defined as LAZ < -2.

**Table G.** Results of subgroup analyses for each outcome at 6 months of age by stunting (height-for-length Z-score < - 2) at enrollment

|  | **Azithromycin** | | | **Placebo** | | |  | |
| --- | --- | --- | --- | --- | --- | --- | --- | --- |
| **Outcome** | **N** | **Mean (SD)** | | **N** | **Mean (SD)** | | **Mean Difference**  **(95 % CI)** | **P-value^1^** |
| **Weight gain (g/day)** | | | |  | | | | |
| Stunted | 1,509 | 19.3 (6.7) | | 1,497 | 19.9 (7.9) | | -0.6 (-1.1 to -0.04) | 0.006 |
| Not stunted | 12,123 | 17.8 (6.1) | | 12,147 | 17.7 (6.1) | | 0.1 (-0.05 to 0.3) |  |
| **Height change (mm/day)** | | | |  | | | | |
| Stunted | 1,509 | 9.6 (2.4) | | 1,497 | 9.6 (3.4) | | 0.01 (-0.2 to 0.2) | 0.89 |
| Not stunted | 12,123 | 7.8 (2.0) | | 12,147 | 7.8 (2.0) | | 0.00 (-0.05 to 0.05) |  |
| **WAZ** |  |  |  |  |  |  |  |  |
| Stunted | 1,509 | -1.5 (1.2) | | 1,497 | -1.6 (1.3) | | -0.02 (-0.1 to 0.05) | 0.32 |
| Not stunted | 12,123 | -0.6 (1.1) | | 12,147 | -0.6 (1.1) | | 0.00 (-0.02 to 0.02) |  |
| **WLZ** |  |  |  |  |  |  |  |  |
| Stunted | 1,509 | -0.9 (1.3) | | 1,497 | -0.8 (1.3) | | -0.06 (-0.1 to 0.03) | 0.07 |
| Not stunted | 12,123 | -0.6 (1.2) | | 12,147 | -0.6 (1.2) | | 0.01 (-0.02 to 0.04) |  |
| **MUAC (cm)** |  |  | |  |  | |  |  |
| Stunted | 1,509 | 13.6 (1.4) | | 1,497 | 13.5 (1.3) | | 0.04 (-0.05 to 0.1) | 0.53 |
| Not stunted | 12,123 | 13.9 (1.2) | | 12,147 | 13.9 (1.2) | | 0.009 (-0.02 to 0.04) |  |
| **LAZ** |  |  | |  |  | |  |  |
| Stunted | 1,509 | -1.3 (1.5) | | 1,497 | -1.4 (1.5) | | 0.06 (-0.04 to 0.16) | 0.04 |
| Not stunted | 12,123 | -0.2 (1.2) | | 12,147 | -0.2 (1.2) | | -0.01 (-0.04 to 0.01) |  |
|  | **Azithromycin** | | | **Placebo** |  | |  |  |
|  | **N with outcome** | | | **N with outcome** | | | **Odds Ratio**  **(95 % CI)** | **P-value^1^** |
| **Underweight^2^** |  | | |  | | |  | 0.99 |
| Stunted | 498 (32.4%) | | | 496 (33.1%) | | | 0.97 (0.83 to 1.13) |  |
| Not stunted | 1,167 (9.6%) | | | 1,204 (9.9%) | | | 0.97 (0.89 to 1.05) |  |
| **Wasted (WLZ)^3^** |  |  | |  |  | |  | 0.86 |
| Stunted | 257 (17.0%) | | | 260 (17.4%) | | | 0.98 (0.81 to 1.18) |  |
| Not stunted | 1,444 (11.9%) | | | 1,453 (12.0%) | | | 1.00 (0.92 to 1.08) |  |
| **Wasted (MUAC)^4^** |  |  | |  |  | |  | 0.84 |
| Stunted | 255 (16.9%) | | | 257 (17.2%) | | | 0.98 (0.81 to 1.19) |  |
| Not stunted | 967 (8.0%) | | | 1,006 (8.3%) | | | 0.96 (0.88 to 1.05) |  |
| **Stunted^5^** |  |  | |  |  | |  | 0.09 |
| Stunted | 470 (31.2%) | | | 507 (33.9%) | | | 0.88 (0.76 to 1.03) |  |
| Not stunted | 665 (5.5%) | | | 641 (5.3%) | | | 1.04 (0.93 to 1.16) |  |

Abbreviations: SD: standard deviation, CI: confidence interval, g, grams, mm, millimeters, WAZ, weight-for-age Z-score, WLZ: weight-for-length Z-score, MUAC: mid-upper arm circumference; ^1^P for interaction for subgroup category by randomized treatment assignment; ^2^Defined as WAZ < -2; ^3^Defined as WLZ < -2; ^4^Defined as MUAC < 12.5 cm; ^5^Defined as LAZ < -2.
