# Supplementary material for: Single-dose azithromycin for infant growth in Burkina Faso: Prespecified secondary anthropometric outcomes from a randomized controlled trial
Source: PLoS Med. 2024 Jan 23;21(1):e1004345. doi: 10.1371/journal.pmed.1004345 (PMC10846702; doi:10.1371/journal.pmed.1004345)
Supplement: S2 Appendix — (PDF) [file pmed.1004345.s002.pdf]

CHATON:
An individual-randomized trial

May 8, 2020

Version 1.8

Statistical Analysis Plan

UCSF Francis I. Proctor Foundation
Centre de Recherche en Sante de Nouna
University of Heidelberg

[redacted]

Introduction

This document (Statistical Analysis Plan, SAP) describes the planned analysis and reporting for the clinical trial, **Post-neonatal early Azithromycin for the Prevention of Mortality in Burkina Faso: An individual-randomized trial.** It includes specifications for the statistical analyses and tables to be prepared for the interim and final Clinical Study Report. This study is a Phase IV clinical trial to compare methods to reduce childhood mortality using mass administration of azithromycin (Pfizer, CAS 83905-01-5) compared to placebo. The content of this Statistical Analysis Plan meets the requirements stated by the US Food and Drug Administration and conforms to the American Statistical Association's Ethical Guidelines.

The following documents were reviewed in preparation of this Statistical Analysis Plan:

- Post-neonatal early Azithromycin for the Prevention of Mortality in Burkina Faso: An individual-randomized trial, Manual of Operations
- Azithromycin for the Prevention of Neonatal, Infant, and Child Mortality in Burkina Faso and Safety Assessment, Proposal
- Statistical Analysis Plan, MORDOR Trial
- ICH Guidance on Statistical Principles for Clinical Trials

The planned analyses described in this SAP will be included in future manuscripts. Exploratory analyses not necessarily identified in this Statistical Analysis Plan may be performed to support the analysis. Unplanned analyses not delineated in this Statistical Analysis Plan will be documented as such in the final Clinical Study Report and manuscripts.

This document will be reviewed prior to the enrollment of patients. All subsequent changes will be indicated by detailed change log in the Appendix.

Contents

1 Summary **4**

1.1 Mortality Trial 4

2 Statistical Analysis **4**

2.1 Planned Analyses 4

2.1.1 Mortality Trial 4

2.2 Interim Monitoring 7

3 Sample Size Considerations **8**

4 Randomization **11**

5 Abbreviations **12**

6 Revision History **13**

1 Summary

1.1 Mortality Trial

The trial profile is given in the Manual of Operations. In the mortality trial, individual children are randomized to two arms: **Azithro** and **Placebo**. The trial is a placebo-controlled, double-masked (i.e. double-blind), individual-randomized clinical trial.

Objective. Determine the efficacy of targeted azithromycin distribution to infants during an early infant healthcare visit (approximately 5th through 8th week of life) on infant mortality. *We hypothesize that infants receiving a single dose of azithromycin during early post-neonatal infancy will have significantly lower all-cause mortality compared to infants receiving placebo.*

2 Statistical Analysis

2.1 Planned Analyses

2.1.1 Mortality Trial

Any child who is enrolled in the trial will be offered placebo or azithromycin in a masked fashion. Any child known to be living at a six-month follow-up visit is counted as alive, any child known to have died at or before the six-month follow-up visit is counted as died, and any other child is counted as missing or lost to follow-up. Deaths that occur between two follow-up timepoints will be assigned person-time in accordance to the midpoint between the two follow up dates (i.e. a death occuring between the baseline and 6-month timepoint will be contribute 3 months of person-time). As a sensitivity analysis, children that were followed up within twelve weeks of six month follow-up date will be analyzed.

Primary Analysis.

The primary analysis will be conducted as **binomial regression** using complementary log-log link. Firth correction will be employed to ensure numerical stabilty. Inference will

be based on the permutation test.

The analysis will be **two-sided**, with a **type I error rate** (alpha) of 0.05.

Statistical considerations.

- Statistical tests will be conducted with Monte Carlo permutation based on the randomization unit. The number of replications will be 10,000, unless the Monte Carlo confidence interval for the P-value includes 0.05. In this case, 10,000,000 will be used, and this fact reported.

- Model adequacy will be checked by examination of residuals or other goodness of fit tests as needed. Inadequate model fit will prompt us to report alternative models.

- Multiple imputation will be used in case of missing baseline covariates (if applicable). Missing outcome variables will be handled by sensitivity analysis and reporting of conditional results.

Supplementary analyses.

The purpose of the supplementary analyses reported in this section is to assess the role of statistical choices and data quality choices in shaping the result.

mortality Fisher exact test

Secondary analyses of mortality.

All secondary analyses will be sharply distinguished from the primary prespecified analysis and will be identified as such. Secondary analyses include outcome variables or planned subsets which contribute either additional insight or address different scientific questions than the primary analysis.

A pre-specified secondary analysis will pool infants randomized in the neonate and individual studies using a binomial regression with complementary log-log link, permuted within study. A pooled sample size of 54,414 infants would have 80% power to detect a 12% reduction in mortality.

age Binomial regression with complementary log-log link, using age at treatment (in weeks)

the number of azithromycin doses received Binomial regression with complementary log-log link at the individual level, clustering on community, using the number of azithromycin doses as the primary predictor

factorial analysis This trial is nested within a community-level randomized trial (ClinicalTrials.gov ID: NCT03676764) that administers twice annual azithromycin or placebo to children ages 1-59 months. A secondary hypothesis is that the effect of early postnatal treatment delivered in this trial could be modified by the broader, community level treatment received. We hypothesize that reduction in mortality due to early infant treatment at the individual level will be larger among children in communities that receive placebo treatment in community-wide distributions compared with children living in communities that also receive active biannual azithromycin treatment. We will test this hypothesis with a binomial regression with complementary log-log link that includes an indicator variable for the individual infant treatment delivered in this trial (our primary analysis), an indicator for community-level treatment (azithromycin versus placebo), and a term for their interaction. We will test for effect modification on the multiplicative and additive scales. We note that to meet enrollment needs for the present trial, children may be enrolled from health facilities outside of the Nouna District study area. These children will contribute to this trial's primary outcome analysis (above), but will not contribute to this factorial analysis as these children will not be recruited from communities randomized in the community-level treatment trial.

Additional secondary outcomes.

Note that participants outside of WHO Child Growth Standards for WAZ (-6 to +5 SD), HAZ (-6 to +6 SD), or WHZ (-5 to +5 SD) will be excluded from all anthropometric analyses.

hospitalization We will report the two-sided 95% confidence interval of the estimated relative risk and tabulate occurrence by age at treatment and gender.

hospitalization and/or deaths Similarly, We will report the two-sided 95% confidence interval of the estimated relative risk and tabulate occurrence by age at treatment and gender.

height, weight, MUAC, WHZ, WAZ, HAZ Weight: comparison of grams/kg/day (growth velocity) between study arms, permuting at the level of the randomization unit; Height: mm/day, analogous to weight. We will report overall change as well as use ANCOVA models comparing outcomes between arms, using baseline as a covariate.

2.2 Interim Monitoring

Efficacy. A single interim analysis conducted at alpha of 0.001 will be conducted. Specifically, the interim analysis will be conducted when full data are available for the first third of patients (six months after the last individual in the first third of patients) or at the end of the first full year, whichever occurs first.

The final analysis would be conducted at alpha 0.049.

Futility. An interim analysis of futility is proposed, to be finalized in consultation with the Data and Safety Monitoring Board. Specifically, we propose to conduct the same regression as in the primary analysis comparing the treatment and control arms using only data from the first year. This analysis will be conducted using simulation. We suggest consideration of the conditional power to detect a 30% effect. If this drops below 10% at the interim analysis, discontinuation of the trial or other changes to the protocol may be made in consultation with the Data and Safety Monitoring Board.

Interim analysis will be executed by the trial biostatistician at the central site.

Adverse events. We will summarize adverse events overall and separately by study arm. We will report adverse events by study arm to the DSMC at interim meetings throughout the trial, but primary investigators will be masked to the arm-stratified results until the primary outcome analysis is unmasked at the end of the trial. We will summarize adverse events (numerator) by children at risk (denominator) and will dis-aggregate and report separately

more common events following best practice recommendations (cited from [LBM+16]). We will estimate the risk difference in adverse events b etween arms and 95% confidence interval for the difference, though we acknowledge that the trial is not necessarily powered to detect differences in adverse events.

3 Sample Size Considerations

We use the following formula:

$$n = \frac{p_0(1-p_0) + p_1(1-p_1)}{(p_1 - p_0)^2}(z_{1-\alpha/2} + z_{1-\beta})^2$$

We assume a mortality probability of 0.04. We also assume a reduction in the probability of mortality, this reduction being 15%. Specifically, the mortality in the azithromycin group is one minus this probability, multiplied by the placebo mortality probability; an effect size expressed by 10% corresponds to multiplying the baseline probability by 1-10% = 1-0.1 = 0.9.

mortality probability assumed 0.04 (over the study period of six months).

effect size assumed to be 15%.

loss to follow-up assumed to be 5%.

The proposed sample size per arm is 16351, for a total of 32702 in both arms.

Sensitivity analysis, 15% effect size

- **mortality rate** 30 per thousand, **loss to follow up** 5%: total enrollment 44024.
- **mortality rate** 47 per thousand, **loss to follow up** 5%: total enrollment 27644.
- **mortality rate** 40 per thousand, **loss to follow up** 10%: total enrollment 34518.

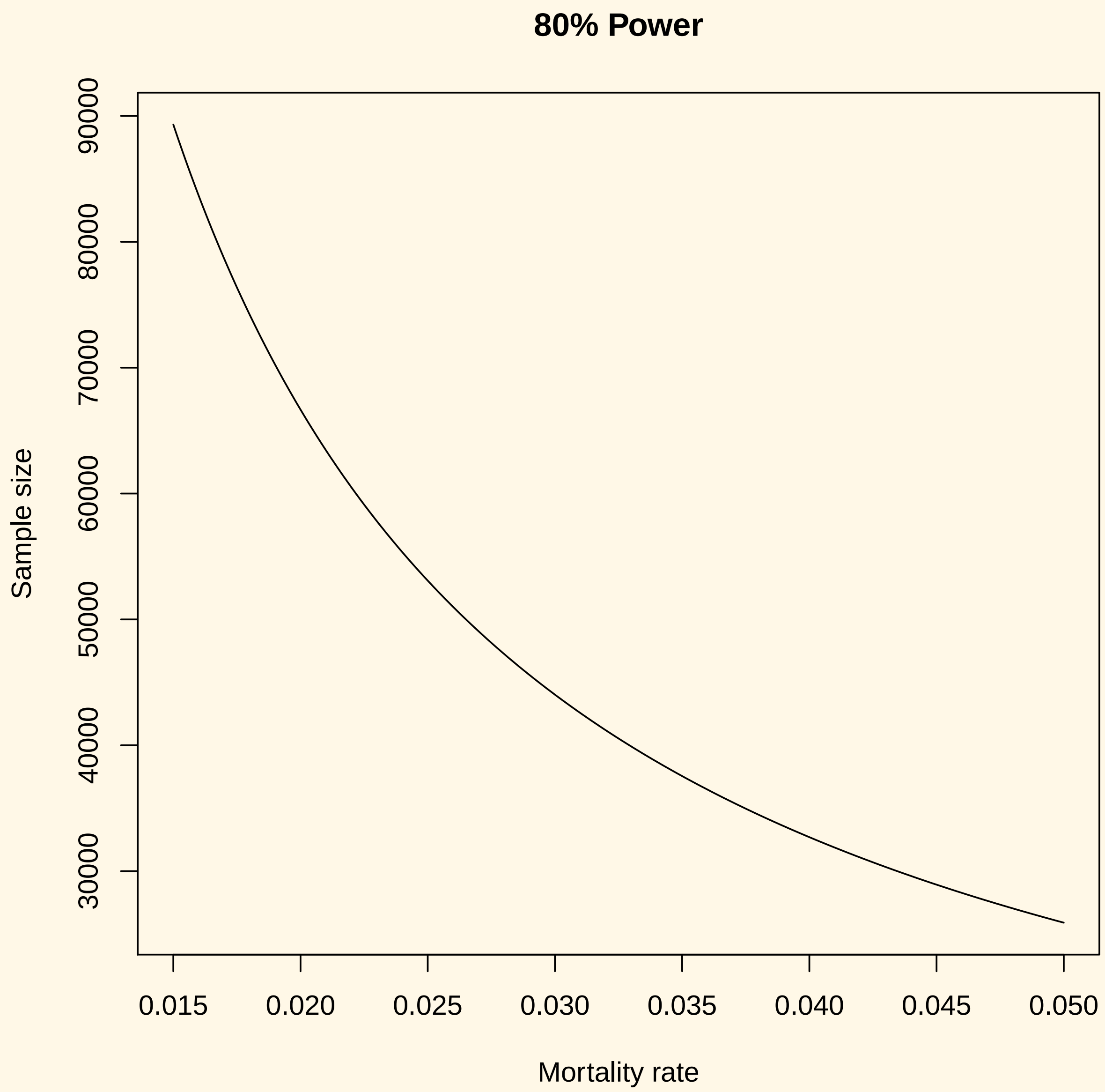
80% Power
Sample size
90000
80000
70000
60000
50000
40000
30000
0.015
0.020
0.025
0.030
0.035
0.040
0.045
0.050
Mortality rate

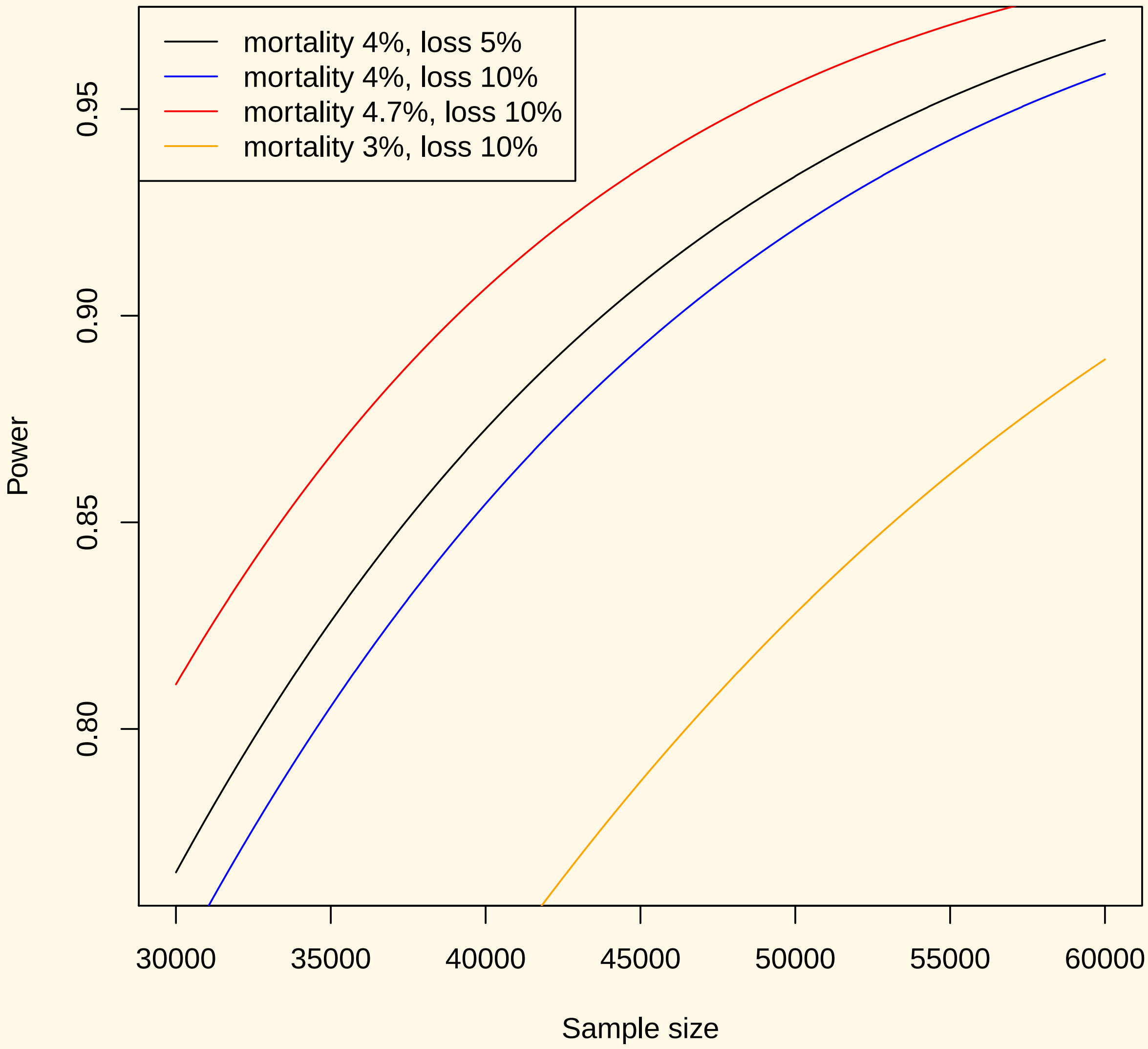

We anticipate 80% power to detect a standardized effect of 0.032 in height or weight (post-test only), based on the sample size and anticipated loss to follow-up.

4 Randomization

The randomization will be conducted using R. The function `sample` with option `replace=FALSE` will be used to conduct the random shuffling. Note that the choice of the random number seed completely determines the randomization. To ensure the integrity of the randomization, we will use the procedure we used for MORDOR/Malawi.

5 Abbreviations

ANCOVA Analysis of covariance

DSMC Data and Safety Monitoring Committee

HAZ height for age Z score

HDSS Health and demographic surveillance system

MUAC Mid upper arm circumference

SAP Statistical Analysis Plan

WAZ weight for age Z score

WHZ weight for height Z score

References

[LBM+16] N. Lineberry, J. A. Berlin, B. Mansi, S. Glasser, M. Berkwits, C. Klem, and et al. Recommendations to improve adverse event reporting in clinical trial publications: a joint pharmaceutical industry/journal editor perspective. *British Medical Journal*, 355, 2016.

6 Revision History

11 Sep 2018 Sample size revision using updated mortality probability.

26 Sep 2018 Sample size formula revision. A pre-specified secondary analysis pooling infants randomized in the neonate and individual studies was added.

5 Oct 2018 Another two pre-specified secondary analyses were added: 1. the number of azithromycin doses received; 2. factorial analysis.

4 Oct 2019 Factorial analysis under section 2.1.1 was updated.

14 Oct 2019 Person-time assignment for events that occur on the same day as enrollment was added in section 2.1.1.

31 Oct 2019 Changed the primary analysis and secondary analysis from logistic regression model to binomial model with a complementary log-log link. Revision to additional secondary outcomes and interim monitoring.

09 Dec 2019 Added adverse events monitoring session under section 2.2 interim monitoring.

09 Dec 2019 Clarified rules for counting deaths and person-time.

16 Jan 2020 Changed title to CHATON.

08 May 2020 Specified WHO standard range for inclusion for anthropometric (child growth) analysis.
